# Hemodynamic goal-directed therapy and postoperative kidney injury: an updated meta-analysis with trial sequential analysis

**DOI:** 10.1186/s13054-019-2516-4

**Published:** 2019-06-26

**Authors:** Mariateresa Giglio, Lidia Dalfino, Filomena Puntillo, Nicola Brienza

**Affiliations:** Anesthesia and Intensive Care Unit, Department of Emergency and Organ Transplantation, University of Bari, Piazza G. Cesare, 11, 70124 Bari, Italy

**Keywords:** Postoperative acute renal injury, Perioperative hemodynamic optimization, High-risk patients, Fluid therapy, Oxygen delivery, Cardiac output

## Abstract

**Background:**

Perioperative goal-directed therapy (GDT) reduces the risk of renal injury. However, several questions remain unanswered, such as target, kind of patients and surgery, and role of fluids and inotropes. We therefore update a previous analysis, including all studies published in the meanwhile, to clarify the clinical impact of this strategy on acute kidney injury.

**Main body:**

Randomized controlled trials enrolling adult patients undergoing major surgery were considered. GDT was defined as perioperative monitoring and manipulation of hemodynamic parameters to reach normal or supranormal values by fluids alone or with inotropes. Trials comparing the effects of GDT and standard hemodynamic therapy were considered. Primary outcome was acute kidney injury, whichever definition was used. Meta-analytic techniques (analysis software RevMan, version 5.3) were used to combine studies, using random-effect odds ratios (OR) and 95% confidence intervals (CI). Trial sequential analyses were performed including all trials and considering only low risk of bias trials. Sixty-five trials with an overall sample of 9308 patients were included. OR for the development of renal injury was 0.64 (95% CI, 0.62–0.87; *p* = 0.0003), with no statistical heterogeneity. Trial sequential analyses and sensitivity analysis including studies with low risk of bias confirmed the main results. A significant decrease in renal injury rate was observed in studies that adopted cardiac output and oxygen delivery as hemodynamic target and that used both fluids and inotropes. The postoperative kidney injury rate was significantly lower in trials enrolling “high-risk” patients and major abdominal and orthopedic surgery.

**Short conclusion:**

The present meta-analysis suggests that targeting GDT to perioperative systemic oxygen delivery, by means of fluids and inotropes, can be the best way to improve renal perfusion and oxygenation in high-risk patients undergoing major abdominal and orthopedic surgery.

**Electronic supplementary material:**

The online version of this article (10.1186/s13054-019-2516-4) contains supplementary material, which is available to authorized users.

## Background

Acute kidney injury (AKI) is an abrupt decrease of renal function, encompassing various etiologies, from pre-renal azotemia to acute tubular necrosis and post-renal obstructive disease. More than one condition may coexist in the same patient, making a uniform definition still a challenge. Recently, a single definition was proposed to be useful for practice and research (i.e., an increase of serum creatinine of 1.5 to 1.9 times baseline or > 0.3 mg/dl, or urine output < 0.5 ml/kg/h for 6 to 12 h [1].

AKI is a well-known complication following surgical procedures [2], independently associated with increased hospital mortality and doubling of hospital costs [3, 4]. Therefore, the prevention of this postoperative complication is of paramount importance.

Perioperative monitoring and manipulation of physiologic hemodynamic parameters to reach adequate cardiac output (CO) and oxygen delivery (DO_2_) (GDT) may decrease the risk of postoperative renal injury [5]. This finding has been confirmed by a subsequent systematic review [6], and in a recent international, web-enabled consensus conference [7], GDT resulted in the strongest recommendation proposed to reduce mortality in patients with or at risk for AKI. Similarly, recent guidelines suggest GDT to prevent the development or worsening of AKI in a perioperative setting (strength of recommendation 2C) [1]. However, interventions to optimize hemodynamics are heterogeneous in targets, timing, design, and technology. Several questions remain unanswered, such as targets, treatment strategies—including the role of fluids and inotropes—and kind of patients and surgeries that can benefit from this approach. In order to clarify these issues, an up-to-date systematic review with meta-analysis and trial sequential analysis (TSA) has been performed.

## Main body

### Materials and methods

#### Eligibility criteria

Studies were searched according to the following eligibility criteria [8]:

##### Types of participants

Adult (age 18 years or over) patients undergoing major surgery were considered. Studies involving mixed population of critically ill, non-surgical patients, or postoperative patients with already established sepsis or organ failure were excluded.

##### Type of intervention

GDT was defined as perioperative monitoring and manipulation of hemodynamic parameters to reach normal or supranormal values by fluids alone or in combination with inotropic therapy, within 8 h after surgery. Studies including late hemodynamic optimization treatment were excluded.

##### Type of comparison

Randomized controlled trials (RCTs) comparing the beneficial and harmful effects of GDT and standard hemodynamic therapy were considered. Standard hemodynamic management was defined as anesthesiologists’ routine administration of fluids and/or inotropic drugs in order to achieve hemodynamic stability, but not aimed to reach physiologic flow-related end-points and not guided by appropriate monitoring. RCTs with no description or no difference in optimization strategy between groups and RCTs with therapy titrated to the same goal in both groups or not titrated to predefined endpoints were excluded.

##### Type of outcome measures

Primary outcome measure was AKI, whichever definition was adopted. A sensitivity analysis was planned, according to the risk of bias of included studies (i.e., RCTs with 5 or 6 green plus, see below). A TSA was performed including all RCTs and low risk of bias trials, to adjust for random error risk.

Several subgroup analyses were planned for the main outcome according to:

- Target. Studies were defined according to the target used in the GDT protocol (indices of pre-load responsiveness, CO or DO_2_, or other indirect indices of DO_2_, such as lactate and venous saturation).
- Treatment. The subset analysis included studies that used fluids alone to optimize hemodynamic status. The other subgroup included studies that used both fluids and inotropes. Moreover, we planned a subgroup analysis including only those RCTs that showed a statistical difference between treatment and control group during the perioperative period in the total amount of starch-based solutions (HES) administered.
- Risk. Studies were split into 2 different subgroups, according to the risk of perioperative morbidity/mortality. Low risk was defined as elective surgery in young, ASA I-II patients. Definition of high risk was based on the need of emergent surgery, and/or elective major surgery in patients with risk criteria defined by perioperative scoring system [9], ASA physical status classification (i.e., ASA III–IV), age > 60 years, and preoperative morbidity.
- Surgery. Studies were divided according to the kind of surgery (i.e., major abdominal, trauma, vascular, cardiac, thoracic, orthopedic).

##### Types of studies

Randomized controlled trials studying perioperative GDT. No language, publication date, or status restrictions were imposed.

#### Information sources

Different search strategies (last update September 2018) were performed to retrieve relevant studies using MEDLINE, The Cochrane Library, and EMBASE databases. No date restriction was applied for MEDLINE and The Cochrane Library databases, while the search was limited to 2007–2018 for EMBASE database [10]. Additional RCTs were searched in The Cochrane Library and in the DARE databases and the reference lists of previously published reviews and retrieved articles, and other data sources were hand-searched in the annual proceedings (2003–2018) of the Society of Critical Care Medicine, the European Society of Intensive Care Medicine, the Society of Cardiovascular Anesthesiologists, the Royal College of Anaesthetists, and the American Society of Anesthesiologists.

#### Search

The search strategies used for MEDLINE, The Cochrane Library, and EMBASE databases are reported in Additional file 1.

#### Study selection

Two investigators (MG, NB) examined at first each title and abstract to exclude clearly irrelevant studies and to identify potentially relevant articles. Other two investigators (FP, LD) independently determined the eligibility of full-text articles retrieved. The names of the author, institution, journal of publication, and results were unknown to the two investigators at this time.

#### Data collection process

Data were independently collected by two investigators (MG, FP) with any discrepancy resolved by re-inspection of the original article. To avoid transcription errors, the data were input into statistical software and rechecked by different investigators (NB, LD).

#### Data items

Data abstraction included patients’ characteristics (age, sex), risk factors, type of hemodynamic GDT (monitoring tools, end-points, therapeutic interventions), type of surgery, incidence (patients who developed postoperative AKI), and definition of postoperative AKI.

#### Risk of bias in individual studies

A domain-based evaluation, as proposed by the Cochrane Collaboration, was used to evaluate the methodological quality of RCTs [11]. This is a two-part tool, addressing seven specific domains (namely, sequence generation, allocation concealment, blinding of participants and personnel, blinding of outcome assessment, incomplete outcome data, selective outcome reporting, and “other issues”) strongly associated with bias reduction [12, 13]. With regard to blinding, studies in which outcome variables were collected by investigators not aware of the intra-operative strategy, as well as studies in which postoperative renal injury was clearly pre-defined, were considered adequately masked.

Each domain in the tool includes one or more specific entries in a “risk of bias” table. Within each entry, the first part of the tool describes what was reported to have happened in the study, in sufficient detail to support a judgment about the risk of bias. The second part of the tool assigns a judgment relating to the risk of bias for that entry. This is achieved by assigning a judgment of “low risk,” “high risk,” or “unclear risk” of bias. After each domain is completed, a “risk of bias summary” figure presenting all of the judgments in a cross-tabulation of study by entry is generated. The green plus indicates a low risk of bias, the red minus indicates a high risk of bias, and the white color indicates an unclear risk of bias. For each study, the number of green plus obtained for every domain was calculated: RCTs with 5 or 6 green plus were considered as low risk of bias studies.

#### Summary measures and planned method of analysis

Meta-analytic techniques (analysis software RevMan, version 5.3 Cochrane Collaboration, Oxford, England, UK) were used to combine studies using odds ratios (OR) and 95% confidence intervals (CI). A statistical difference between groups was considered to occur if the pooled 95% CI did not include 1 for the OR. An OR less than 1 favored GDT when compared with the control group. Two-sided *p* values were calculated. A random-effects model was chosen for all analyses. Statistical heterogeneity and inconsistency were assessed by using the *Q* and *I*^2^ tests, respectively [14, 15]. When the *p* value of the *Q* test was < 0.10 and/or the *I*^2^ was > 40%, heterogeneity and inconsistency were considered significant [16]. When significant heterogeneity and inconsistency were found, the most heterogeneous study on the basis of the forest plot was removed and the analysis was re-done. Two TSA were performed including all trials and only low risk of bias RCTs. The information size and monitoring boundary were calculated anticipating a 2% relative risk reduction in postoperative AKI with GDT. We set risk of type I at 5% and power at 95%.

## Results

### Study selection

The search strategies identified 3304 (MEDLINE), 9992 (Cochrane Library), and 3492 (EMBASE) articles. Fifteen articles were identified through other sources (congress abstracts, reference lists). After initial screening and subsequent selection, a pool of 126 potentially relevant RCTs was identified. The subsequent eligibility process (Additional file 2: Figure S1) excluded 61 articles and, therefore, 65 [17–81] RCTs with a total sample of 9308 patients were considered for the analysis.

### Study characteristics

All included articles evaluated the effects of hemodynamic GDT on morbidity (including AKI) as primary or secondary outcome and had a population sample of adult surgical patients, undergoing both elective and emergent procedures (Additional file 7: Table S1). The studies were performed in Australia, USA, Europe, Canada, Brazil, China, Africa, and India from 1991 to 2018 and were all published in English.

Data concerning morbidity/mortality risk definition, population, type of surgery, monitoring tools, and targets are presented in Additional file 7: Table S1. The risk of bias assessment for each trial is showed in Additional file 8: Table S2. Out of 65 studies, 29 enrolled “high-risk” patients. In 26 studies, the treatment group received only fluids (crystalloids, gelofusine, HES) and/or blood, while in 39 studies, optimization was obtained both with fluids (crystalloids and/or colloids and/or blood) and inotropes (dopamine, dobutamine, dopexamine, or epinephrine) with vasodilators. In one study [74], either dopexamine or epinephrine was administered in the treatment group; both groups were pooled together for the purpose of the analysis. In one study [42], one treatment group received dopamine not targeted to hemodynamic end-point, and therefore, this group was not considered for the purpose of the analysis.

### Quantitative data synthesis

**Fig. 1:**
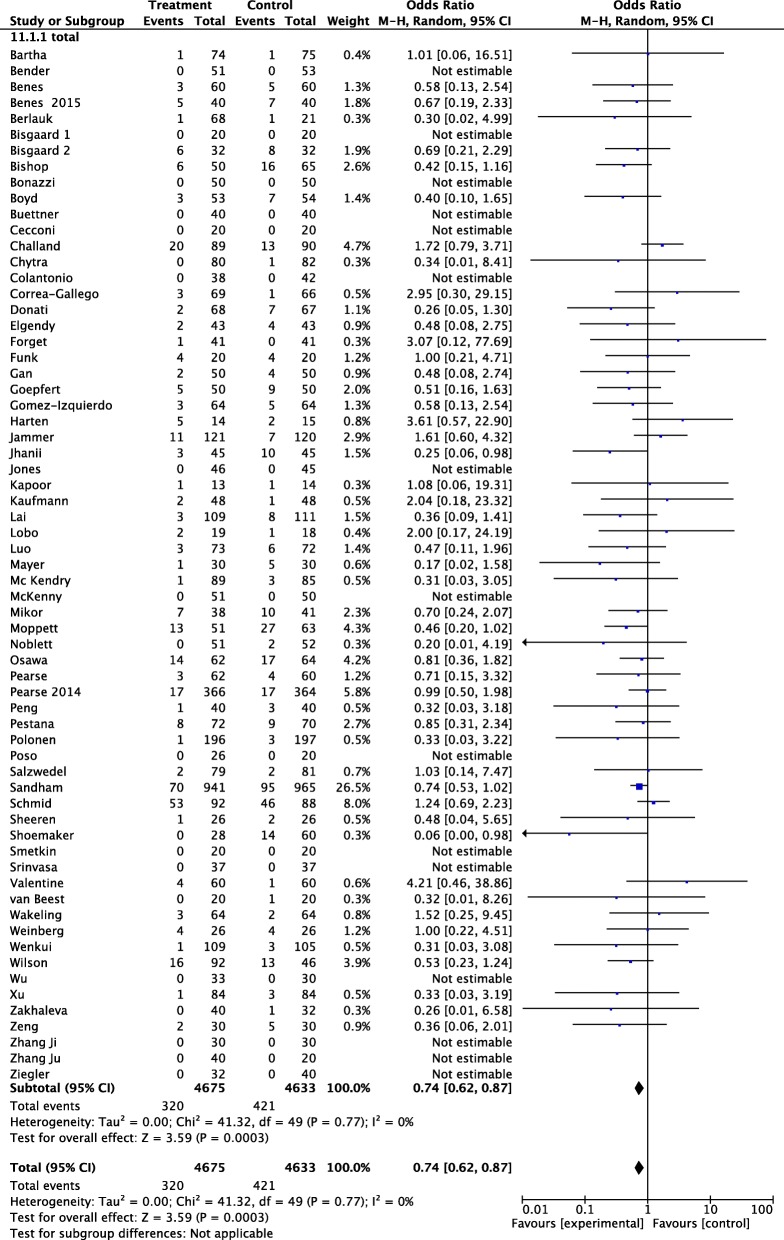
Forest plot for postoperative acute kidney injury (AKI) (defined as the proportion of patients who developed postoperative worsening of renal function, whichever definition was used). Size of squares for odds ratio reflects weight of trial in pooled analyses. Horizontal bars represent 95% confidence intervals

Among the 9308 patients randomized in the 65 included studies, 741 developed AKI. Of these, 421 had been randomized to control group (9.01%) and 320 (6.84%) to GDT. The pooled OR and 95% CI for the development of AKI were 0.64, 95% CI 0.62–0.87; *p* = 0.0003 (Fig. 1). No statistical heterogeneity was detected.

**Fig. 2:**
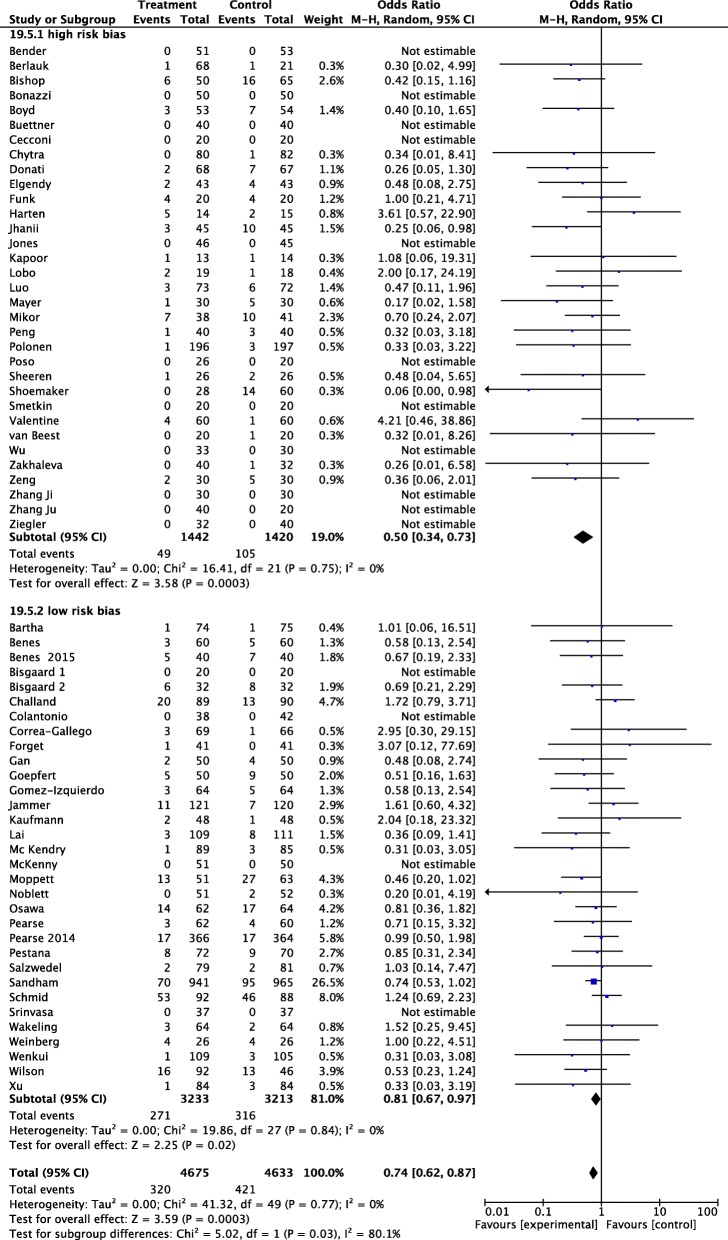
Forest plot for postoperative acute kidney injury (AKI) (defined as the proportion of patients who developed postoperative worsening of renal function, whichever definition was used by the authors of the included studies). Studies were split in high and low risk of bias, according to a domain-based evaluation, as proposed by the Cochrane Collaboration. RCTs with 5 or 6 green plus were considered as having an overall low risk of bias (see text for details). Size of squares for odds ratio reflects weight of trial in pooled analyses. Horizontal bars represent 95% confidence intervals

TSA confirmed the main result: the cumulative *z* curve crosses the O’Brien-Fleming boundaries, and the meta-analysis can be declared as conclusive with regard to the effect of GDT on AKI (Additional file 3: Figure S2). The sensitivity analyses, including only low risk of bias RCTs, confirmed the main analysis, with an OR 0.81, 95% CI 0.6–0.97 (Fig. 2). TSA considering only low risk of bias RCTs shows the same conclusion: the cumulative *z* curve crossed the O’Brien-Fleming boundaries, indicating firm evidence that GDT reduces postoperative AKI (Additional file 4: Figure S3).

**Fig. 3:**
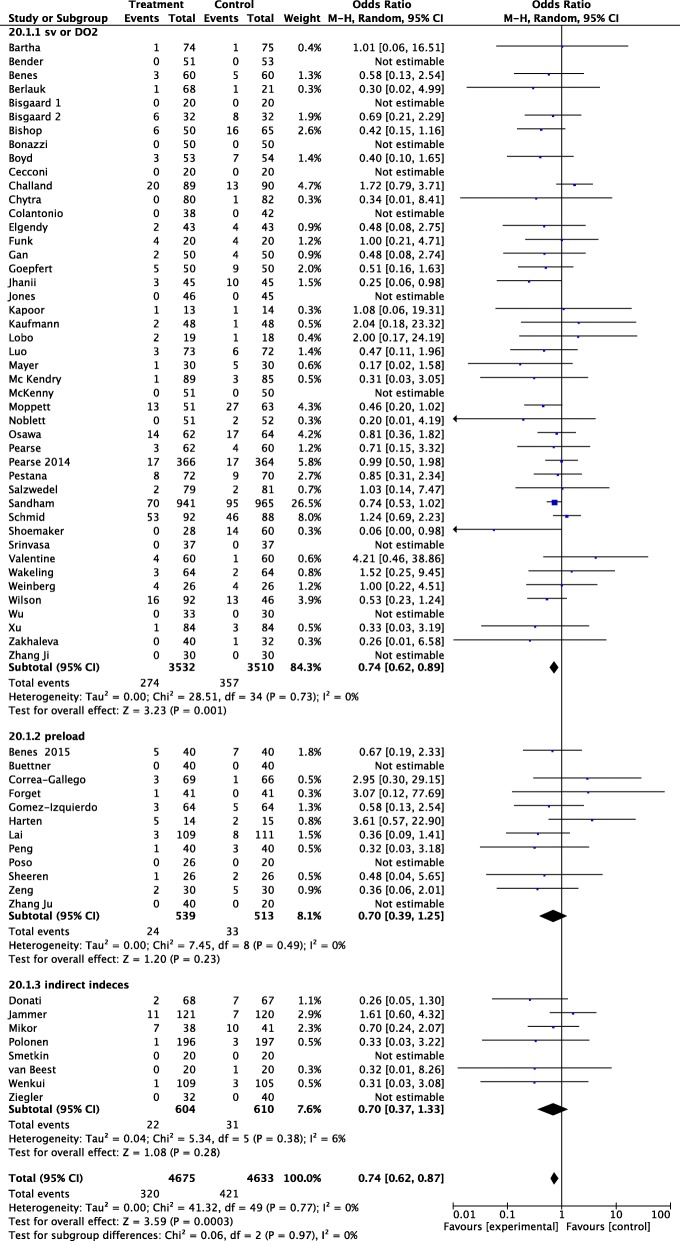
Forest plot for postoperative acute kidney injury (AKI) (defined as the proportion of patients who developed postoperative worsening of renal function, whichever definition was used). Studies were defined according to the target used in the GDT protocol (stroke volume and oxygen delivery or other indexes of oxygen delivery, indices of fluid-responsiveness, such as pulse pressure variation or stroke volume variation, and mixed venous oxygen saturation or other indirect indexes of oxygen delivery such as lactate). Size of squares for odds ratio reflects weight of trial in pooled analyses. Horizontal bars represent 95% confidence intervals

The subset analysis including studies using CO or DO_2_ as hemodynamic target showed a significant reduction in AKI (OR 0.64, 95% CI 0.62 to 0.89, *p* = 0.001), while OR of studies that used preload indices or indirect indices as lactate did not reach statistical significance (Fig. 3).

**Fig. 4:**
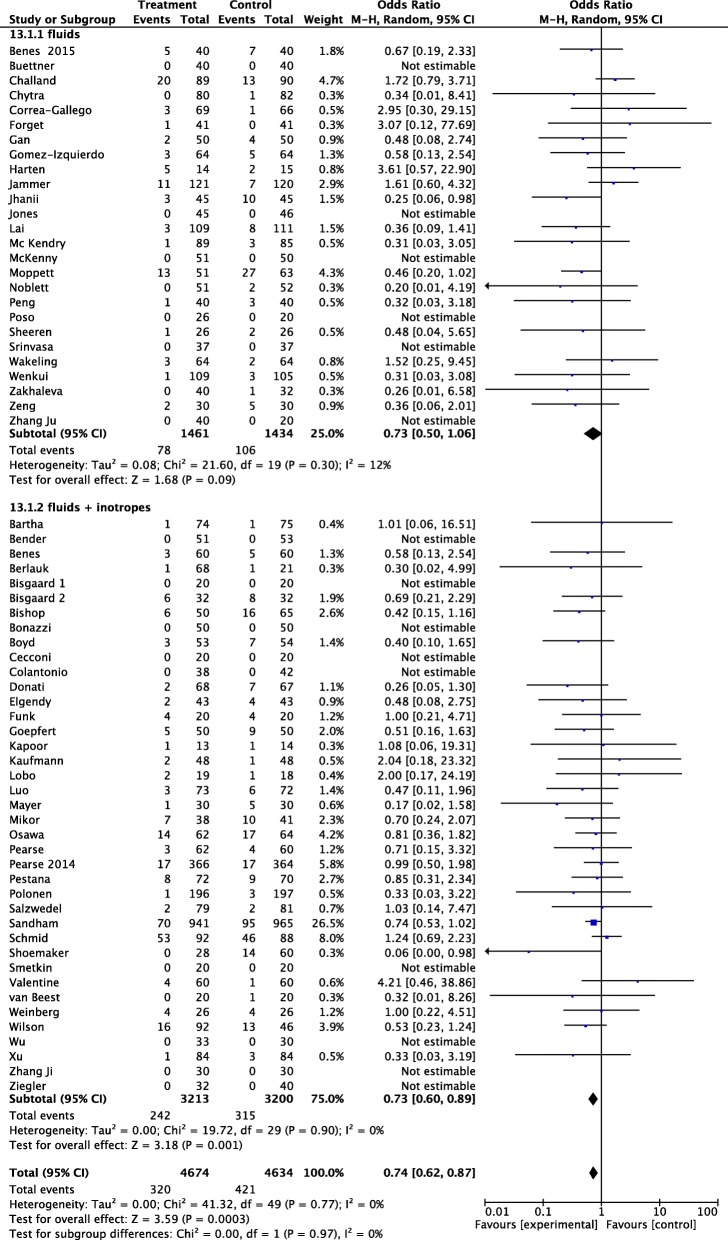
Forest plot for postoperative acute kidney injury (AKI) (defined as the proportion of patients who developed postoperative worsening of renal function, whichever definition was used). Studies were split into trials that used fluids alone or both fluids and inotropes. Size of squares for odds ratio reflects weight of trial in pooled analyses. Horizontal bars represent 95% confidence intervals

**Fig. 5:**
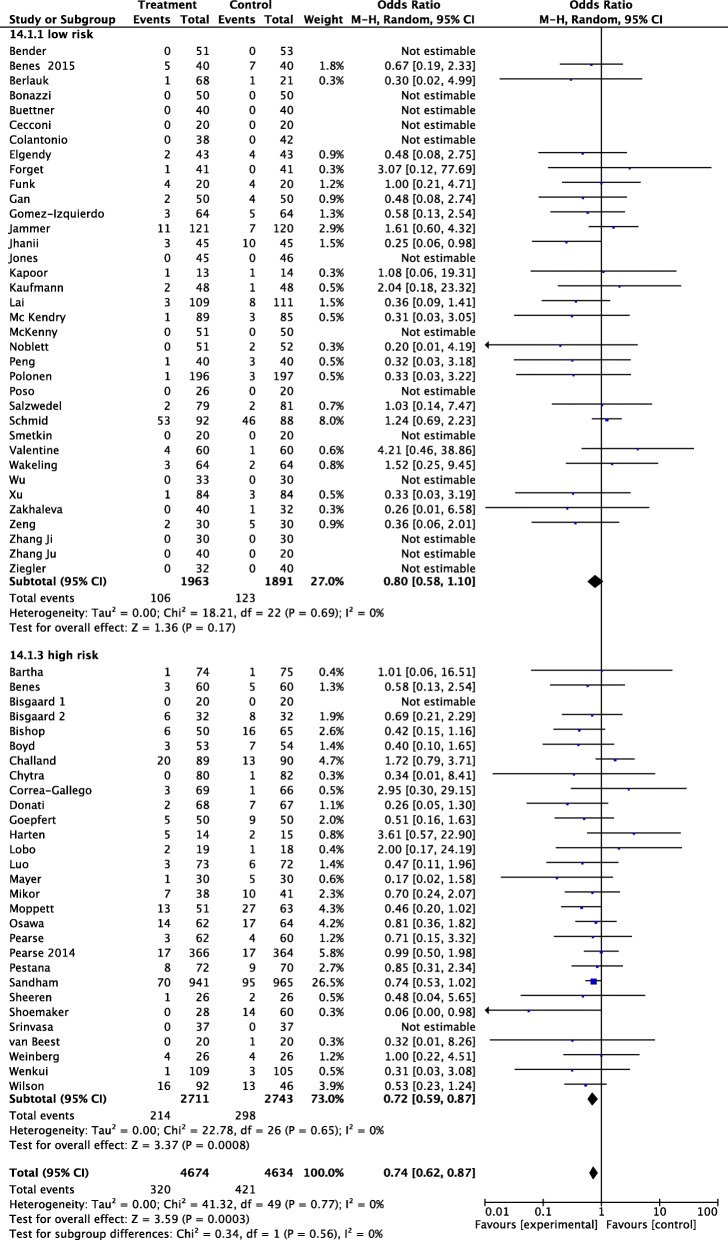
Forest plot for postoperative acute kidney injury (AKI) (defined as the proportion of patients who developed postoperative worsening of renal function, whichever definition was used by the authors of the included studies). Studies were split into 2 different subgroups, according to the risk of perioperative morbidity/mortality. Low risk was defined as elective surgery in young, ASA I-II patients. Definition of high risk was based on the need of emergent surgery, and/or elective major surgery in patients with risk criteria defined by perioperative scoring system, ASA physical status classification (i.e., ASA III–IV), age > 60 years, and preoperative morbidity. Size of squares for odds ratio reflects weight of trial in pooled analyses. Horizontal bars represent 95% confidence intervals

Subgroup analysis showed that fluid administration alone did not reduce AKI, while a significant decrease in AKI rate was observed in patients receiving both fluids and inotropes (OR 0.73 95% CI 0.60 to 0.89, *p* = 0.001) (Fig. 4). The subgroup analysis including RCTs that reported a statistical difference between treatment and control group in total amount of HES given during the perioperative period showed no difference in postoperative AKI (OR 0.85 95% CI 0.64 to 1.12, *p* = 0.24; *I*^2^ 0%, 33 RCTs, 3871 patients) (Additional file 5: Figure S4). Postoperative AKI rate was significantly lower in studies enrolling “high-risk” patients (OR, 0.72; 95% CI 0.59 to 0.87, *p* = 0.0008); in low-risk patients, no difference in AKI was observed (Fig. 5).

Subgroup analyses based on the kind of surgery yielded significant differences in AKI between treatment and control groups for patients undergoing major abdominal and orthopedic surgery (OR 0.78; 95% CI 0.63–0.96, *p* = 0.03, and OR 0.51; 95% CI 0.27–0.96, *p* = 0.04, respectively), while no difference was observed in other types of surgical procedures (Additional file 6: Figure S5). Excluding the heaviest trial [63] did not significantly change main and subgroup analyses, while the sensitivity analysis, including only low risk of bias RCTs, yielded a not significant reduction in AKI.

## Discussion

The present meta-analysis demonstrates that the incidence of postoperative AKI is reduced by GDT: this significant reduction was confirmed in the sensitivity analysis enrolling only low risk of bias trials. TSA, performed to unmask false-positive results [82], confirms the robustness of the data, since the number of patients enrolled (9308 patients) is very near to the required information size (9668 patients) to reach a definite conclusion. In order to reduce AKI incidence, a strategy that is guided by CO and DO_2_ should be adopted, using both fluids and inotropes. Patients who more benefit from this approach are high-risk patients undergoing abdominal or orthopedic surgery.

The role of kidney hypoperfusion and hypoxia has been recently underlined as a key pathogenetic event promoting postoperative AKI [83]. Tissue hypoxia triggers a vicious cycle of inflammation, peritubular capillary narrowing, impaired renal autoregulation, oxidative stress, apoptosis, and necrosis [84]. Protection against hypoperfusion mainly relies on maintaining adequate intravascular volume and organ perfusion pressure. Several evidence confirm this approach, and a very recent trial [85] suggests that an “individualized” blood pressure control, with a protocolized hemodynamic algorithm to guide fluid delivery and to maximize stroke volume, could reduce the incidence of AKI.

Other authors [86] confirmed that intraoperative lactic acidosis or vasopressor requirement precedes subsequent AKI development and that failure to achieve preoperative DO_2_ is significantly associated with the increase of postoperative creatinine. Interestingly, AKI was not prevented by GDT or standard care after lactic acidosis developed or vasopressors were required. Taking together, these data suggest that both lactic acidosis and hypotension may be late indicators of a reduction of renal perfusion pressure, and that, in order to avoid AKI, the best goal is to maintain an individualized DO_2_. Basing on this rationale, fluid resuscitation is crucial to maintain CO and renal blood flow. GDT allows a timelier fluid replacement strategy in patients who need it, avoiding at the same time excessive fluid loading in patients that do not [5]. In all GDT protocols, however, fluid resuscitation is only the first step. In patients who cannot achieve adequate DO_2_, inotropes are necessary, acting in a synergistic manner with fluids, since GDT fluid therapy allows optimal use of inotropic drugs, and inotropic drugs reduce the risk of fluid overload, optimizing CO [5]. Our results further reinforce these figures, since GDT guided by CO and DO_2_ as hemodynamic target, with fluids and inotropes, shows a significant reduction in AKI.

Recent evidence suggests that the type of fluid may be critical in determining AKI [87]. Several concerns about renal toxicity of HES solutions have been raised, and their safety in surgical patients is still under debate [88, 89]. We tried to investigate the effect of GDT adopting HES solutions: the subgroup analysis including only RCTs that showed a statistical difference between treatment and control group during the perioperative period in the total amount of HES administered did not find any statistical difference in AKI incidence. In most studies, HES were used both in intervention and control groups. Therefore, in order to explore the association between AKI and HES, we tried to select papers on the basis of a significant difference in the amount given. Colloids seem neither to benefit nor to harm AKI if given within an individualized, timely fluid “replacement” strategy. Interestingly, a very recent RCT [90] reached the same results. However, no clear conclusion can be drawn, since, paradoxically, colloids might harm the kidney in the context of a beneficial GDT effect. Further trials are needed to investigate the effect of starch solution on AKI in surgical patients.

GDT significantly reduced the incidence of postoperative AKI in high-risk patients that included aged people, ASA III–IV, with increased risk of mortality and morbidity due to reduced cardiovascular reserve, undergoing high-risk procedures with increased risk of blood loss and/or fluid shift. These characteristics are all well-known risk factors for postoperative renal injury [91]. Therefore, it is logical to argue that this category of frail patients would more benefit from GDT to improve systemic oxygenation and to maintain organ perfusion.

The subgroup analysis on surgeries showed that GDT significantly reduced AKI after abdominal and orthopedic procedures, while no effect was seen in other surgeries. Surgical stress may increase oxygen demand up to 40% in major abdominal surgery [92]. Moreover, major abdominal surgery can cause an increase of intra-abdominal pressure, linked to an increase in capillary permeability and interstitial fluid accumulation or to a diminished abdominal wall compliance that, in turn, causes intrarenal vascular congestion with a reduction in renal perfusion [83]. On the other side, orthopedic patients include often very aged people with severe co-morbidities (i.e., hypertension, renal failure, diabetes) that easily expose them to an increased risk of postoperative AKI [93]. Recent findings suggest that advanced age, hypertension, general anesthesia, and low intraoperative arterial pressure are all risk factors for AKI after joint replacement surgery [94]. Therefore, a strategy aimed to maintain CO seems reasonable to protect against AKI, at least in these surgical settings. No definite conclusion on other surgeries could be drawn, since the low number of included trials in other subgroup analysis is not sufficient to detect any effect, precluding any definite conclusions.

This study has a number of limitations. No attempt was made to correct for the type or quantity of fluids or inotropes given, because they are inconsistently reported in the literature and have a demonstrable wide variability in their dosing across studies. Moreover, the included studies vary in terms of hemodynamic monitoring, the goals, and the timing of intervention: this could have introduced a relatively high clinical heterogeneity, although the results remain consistent across a number of subgroups and sensitivity analyses.

Additional well-designed RCTs are necessary to reach the target of an “individualized” GDT, for example by better defining renal risk, or preoperative cardiological performance, the amount of fluid, and the dose of vasoactive administered, using accepted and uniform definitions, as well as a consistent AKI definition, like KIDGO proposes. Recent trials [95, 96] gave some interesting insight on this approach, suggesting that an implementation of the KDIGO guidelines, including hemodynamic optimization, reduced the frequency and severity of postoperative AKI in high-risk patients, identified by urinary biomarkers.

## Conclusions

This up-to-date meta-analysis, within the limitations of existing data, the high clinical heterogeneity and the analytic approaches used, confirms that GDT significantly reduces postoperative AKI. The result is reinforced by TSA and considering only low risk of bias trials. Moreover, it suggests that targeting GDT to perioperative systemic DO_2_, by means of fluid and inotropes, is the simplest way to improve renal perfusion, at least in high-risk patients undergoing abdominal or orthopedic procedures.

## Additional files

Additional file 1: Search strategies. (DOCX 9 kb)

Additional file 2: **Figure S1.** Flow chart summarizing the studies selection procedure for the meta-analysis. (TIFF 1519 kb)

Additional file 3: **Figure S2.** Trial sequential analysis of postoperative acute kidney injury, including all trials. A diversity adjusted information size of 9668 patients was calculated using *α* = 0.05 (two-sided), *β* = 0.20 (power 95%), an anticipated relative risk reduction of 2%, and an event proportion of 9% in the control arm. The blue cumulative *z* curve was constructed using a random effects model. (TIFF 3072 kb)

Additional file 4: **Figure S3.** Trial sequential analysis of postoperative acute kidney injury, including only low risk of bias trials. A diversity adjusted information size of 9668 patients was calculated using α = 0.05 (two-sided), *β* = 0.20 (power 95%), an anticipated relative risk reduction of 2%, and an event proportion of 9% in the control arm. The blue cumulative *z* curve was constructed using a random effects model. (TIFF 3072 kb)

Additional file 5: **Figure S4.** Forest plot for postoperative acute kidney injury (AKI) (defined as the proportion of patients who developed postoperative worsening of renal function, whichever definition was used) including only those RCTs that showed a statistical difference between treatment versus control group during the perioperative period in the total amount of starch-based solutions (HES) administered. Size of squares for odds ratio reflects weight of trial in pooled analyses. Horizontal bars represent 95% confidence intervals. (TIFF 3072 kb)

Additional file 6: **Figure S5.** Forest plot for postoperative acute kidney injury (AKI) (defined as the proportion of patients who developed postoperative worsening of renal function, whichever definition was used). Studies were divided according to the kind of surgery (i.e., major abdominal, vascular, cardiac, thoracic, orthopedic, trauma surgery). Size of squares for odds ratio reflects weight of trial in pooled analyses. Horizontal bars represent 95% confidence intervals. (EPS 1847 kb)

Additional file 7: **Table S1.** Data concerning RCTs morbidity/mortality risk definition, population and type of surgery, tools and target used. (DOCX 23 kb)

Additional file 8: **Table S2.** The risk of bias assessment for each trial, according to the Cochrane domain-based evaluation. (DOCX 17 kb)

## Data Availability

All data generated or analyzed during this study are included in this published article [and its supplementary information files].

## Abbreviations

AKI: Acute kidney injury
ASA: American Society of Anesthesiologists
CI: Confidence intervals
CO: Cardiac output
DO_2_: Oxygen delivery
GDT: Goal-directed therapy
HES: Starch-based solutions
OR: Odds ratios
RCTs: Randomized controlled trials
TSA: Trial sequential analysis
