# Supplementary material for: Hemodynamic goal-directed therapy and postoperative kidney injury: an updated meta-analysis with trial sequential analysis
Source: Crit Care. 2019 Jun 26;23:232. doi: 10.1186/s13054-019-2516-4 (PMC6593609; doi:10.1186/s13054-019-2516-4)
Supplement: Supplementary file 1 — Search strategies. (DOCX 9 kb) [file 13054_2019_2516_MOESM1_ESM.docx]

**Additional file 1** Search Strategies

For the MEDLINE database, the Cochrane highly sensitive search strategy was used:

# 1 randomized controlled trial [pt]

# 2 controlled clinical trial [pt]

# 3 randomized [tiab]

# 4 placebo [tiab]

# 5 clinical trial as topic[mesh:noexp]

# 6 randomly [tiab]

# 7 trial [ti]

# 8 # 1 OR #2 OR #3 OR #4 OR # 5 OR #6 OR #7

# 9 animals [mh] not (humans [mh] and animals [mh])

# 10 # 8 NOT #9

# 11 surgery [mh]

# 12 surgery [tiab]

# 13 surgery [sh]

# 14 surgery [mh] OR surgery [tiab] OR surgery [sh]

# 15 goal directed [tiab] OR goal directed [sh] OR goal directed [mh]

# 16 goal oriented [tiab] OR goal oriented [sh] OR goal oriented [mh]

# 17 goal target [tiab] OR goal target [sh] OR goal target [mh]

# 18 cardiac output [tiab] OR cardiac output [mh] OR cardiac output [sh]

# 19 cardiac index [tiab] OR cardiac index [mh] OR cardiac index [sh]

# 20 oxygen delivery [tiab] OR oxygen delivery [mh] OR oxygen delivery [sh]

# 21 oxygen consumption [tiab] OR oxygen consumption[mh]

# 22 cardiac volume[tiab] OR cardiac volume[mh]

# 23 stroke volume[tiab] OR stroke volume[mh] OR stroke volume [sh]

# 24 fluid therapy [tiab] OR fluid therapy [mh]

# 25 fluid loading [tiab] OR fluid loading[mh] OR fluid loading[sh]

# 26 fluid administration [tiab] OR fluid administration [mh] OR fluid administration[sh]

# 27 optimization[tiab] OR optimization[mh] OR optimization[sh]

# 28 optimisation[tiab] OR optimisation[mh] OR optimisation[sh]

# 29 supranormal[tiab] OR supranormal[mh] OR supranormal[sh]

# 30 sopranormal[tiab] OR sopranormal[mh] OR sopranormal[sh]

# 31 # 15 OR # 16 OR #17 OR # 18 OR # 19 OR # 20 OR #21 OR # 22 OR # 23 OR # 24 OR # 25 OR # 26 OR # 27 OR #28 OR # 29 OR #30

# 30 # 10 AND # 14 AND # 31.

For Embase, the following search strategy was used:

# 1 random$

# 2 randomized controlled trial/exp

# 3 cross-over procedures/exp

# 4 double blind procedures/exp

# 5 single blind procedures/exp

# 6 factoral$

# 7 crossover$

# 8 cross over$

# 9 cross-over$

# 10 placebo$

# 11 assign$

# 12 allocat$

# 13 volunteer$

# 14 # 1 OR #2 OR #3 OR #4 OR #5 OR #6 OR #7 OR #8 OR #9 OR #10 OR #11 OR #12 OR #13

# 15 surgery

# 16 ('surgery'/exp/mj OR 'surgery')

# 17 'surgery'/syn

# 18 #15 OR #16 OR #17

# 19 ('heart'/exp/mj OR 'heart') AND output

# 20 'heart output'/exp/mj OR 'heart output')

# 21 “goal directed”

# 22 “goal oriented”

# 23 “goal target”

# 24 'heart index'/exp/mj OR 'heart index'

# 25 ('heart stroke volume'/exp/mj OR 'heart stroke volume'

# 26 ('oxygen consumption'/exp/mj OR 'oxygen consumption')

# 27 “oxygen delivery”

# 28 “fluid therapy”/exp

# 29 “fluid loading”/exp

# 30 “fluid administation”/exp

# 31 “supranormal”

# 32 “sopranomal”

# 33 “optimization”

# 34 “optimization”

# 35 # 19 OR # 20 OR #21 OR # 22 OR # 23 OR # 24 OR # 25 OR #26 OR # 27 OR #28 OR # 29 OR #30 OR #31 OR #32 OR #33 OR #34

# 36 #14 AND #18 AND #35.

For the Cochrane Library database, the following search strategy was used:

#1 MeSh descriptor Surgery explode all trees

# 2 MeSh descriptor Specialities, Surgery explode all trees

# 3 surgical*

# 4 surgery*

# 5 # 1 OR #2 OR #3 OR # 4

# 6 MeSh descriptor Cardiac Output explode all trees

# 7 Cardiac near output*

# 8 Cardiac near index*

# 9 Cardiac near volume*

# 10 MeSh descriptor Oxygen Delivery explode all trees

# 11 Oxygen near delivery*

# 12 MeSh descriptor Oxygen Consumption explode all trees

# 13 Oxygen near consumption*

# 14 MeSh descriptor Supranormal explode all trees

# 15 Oxygen near supranormal*

# 16 Oxygen near sopranormal*

# 17 MeSh descriptor Stroke Volume explode all trees

# 18 Stroke near volume*

# 19 MeSh descriptor Fluid Therapy explode all trees

# 20 Fluid near therapy*

# 21 Fluid near administration*

# 22 Fluid near loading*

# 23 MeSh descriptor Goal Directed explode all trees

# 24 Goal near directed*

# 25 Goal near oriented*

# 26 Goal near targeted*

# 27 # 6OR # 7 OR # 8 OR # 9 OR # 10 OR #11 OR # 12 OR # 13 OR # 14 OR # 15 OR # 16 OR #17 OR # 18 OR # 19 OR # 20 OR #21 OR # 22 OR # 23 OR # 24 OR # 25 OR # 26

# 28 # 5 AND # 27.
