# Supplementary figures and images for: Hemodynamic goal-directed therapy and postoperative kidney injury: an updated meta-analysis with trial sequential analysis
Source: Crit Care. 2019 Jun 26;23:232. doi: 10.1186/s13054-019-2516-4 (PMC6593609; doi:10.1186/s13054-019-2516-4)

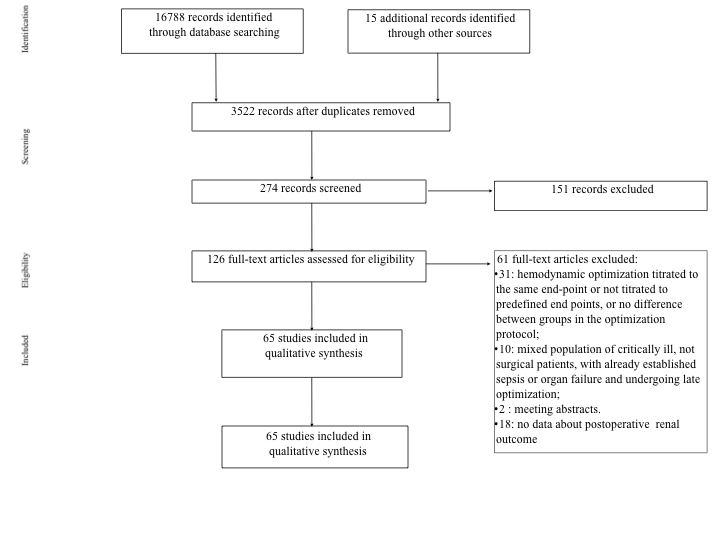

Supplement: Supplementary file 2 — Figure S1. Flow chart summarizing the studies selection procedure for the meta-analysis. (TIFF 1519 kb) [file 13054_2019_2516_MOESM2_ESM.tiff]

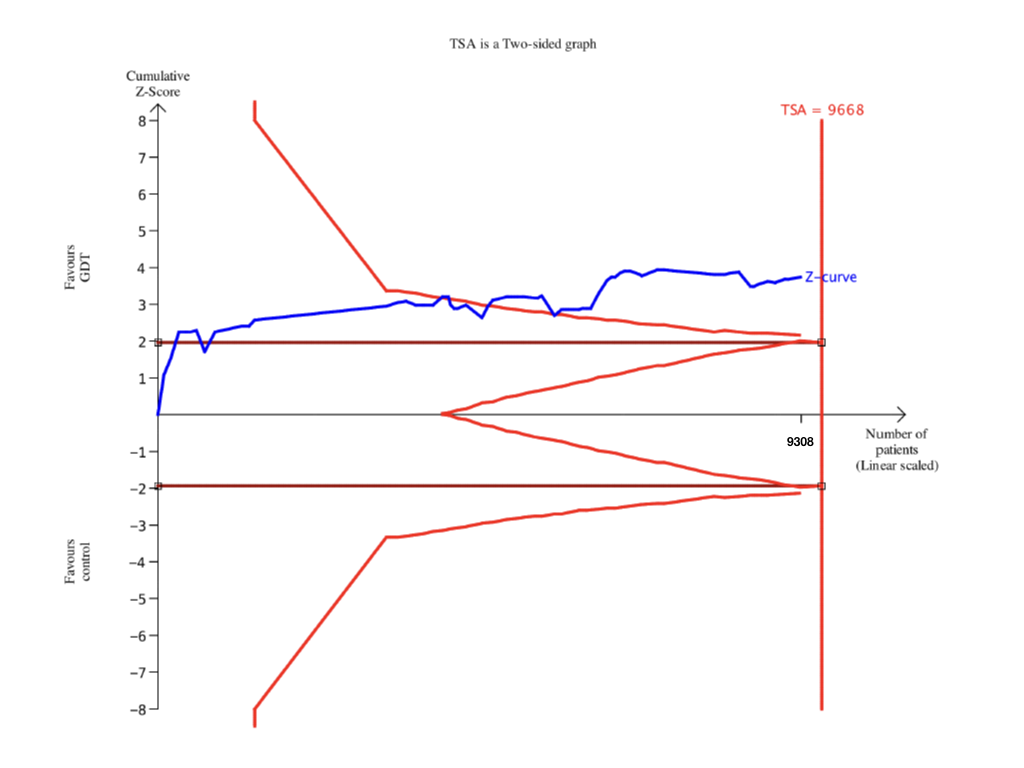

Supplement: Supplementary file 3 — Figure S2. Trial sequential analysis of postoperative acute kidney injury, including all trials. A diversity adjusted information size of 9668 patients was calculated using α = 0.05 (two-sided), β = 0.20 (power 95%), an anticipated relative risk reduction of 2%, and an event proportion of 9% in the control arm. The blue cumulative z curve was constructed using a random effects model. (TIFF 3072 kb) [file 13054_2019_2516_MOESM3_ESM.tiff]

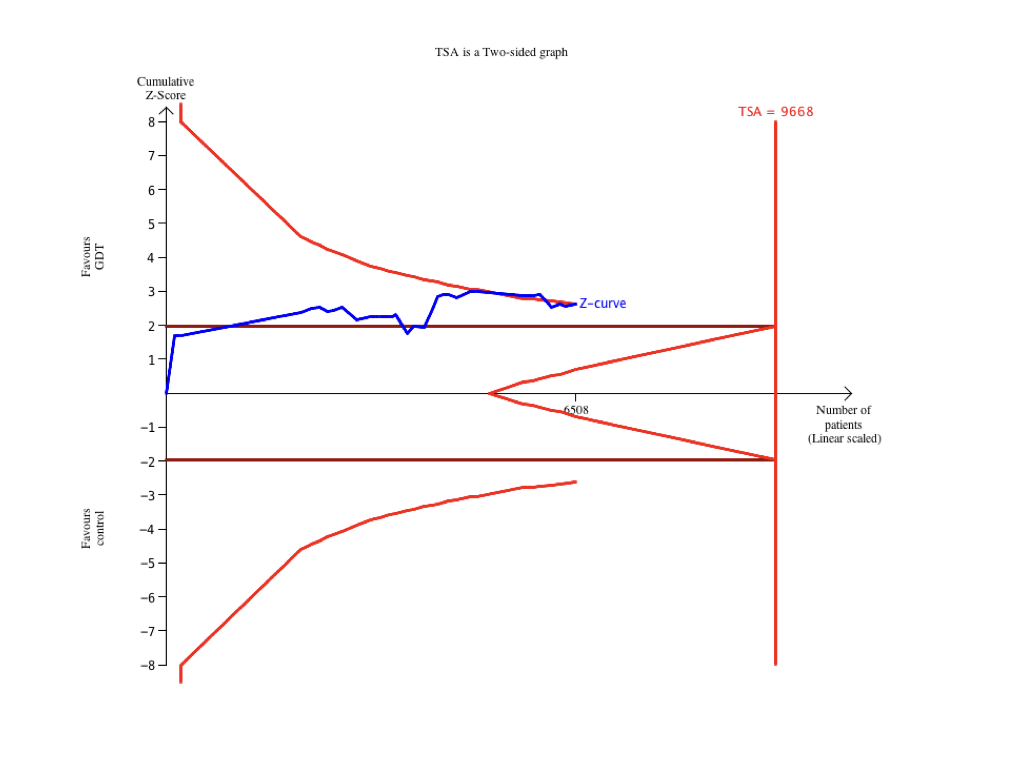

Supplement: Supplementary file 4 — Figure S3. Trial sequential analysis of postoperative acute kidney injury, including only low risk of bias trials. A diversity adjusted information size of 9668 patients was calculated using α = 0.05 (two-sided), β = 0.20 (power 95%), an anticipated relative risk reduction of 2%, and an event proportion of 9% in the control arm. The blue cumulative z curve was constructed using a random effects model. (TIFF 3072 kb) [file 13054_2019_2516_MOESM4_ESM.tiff]

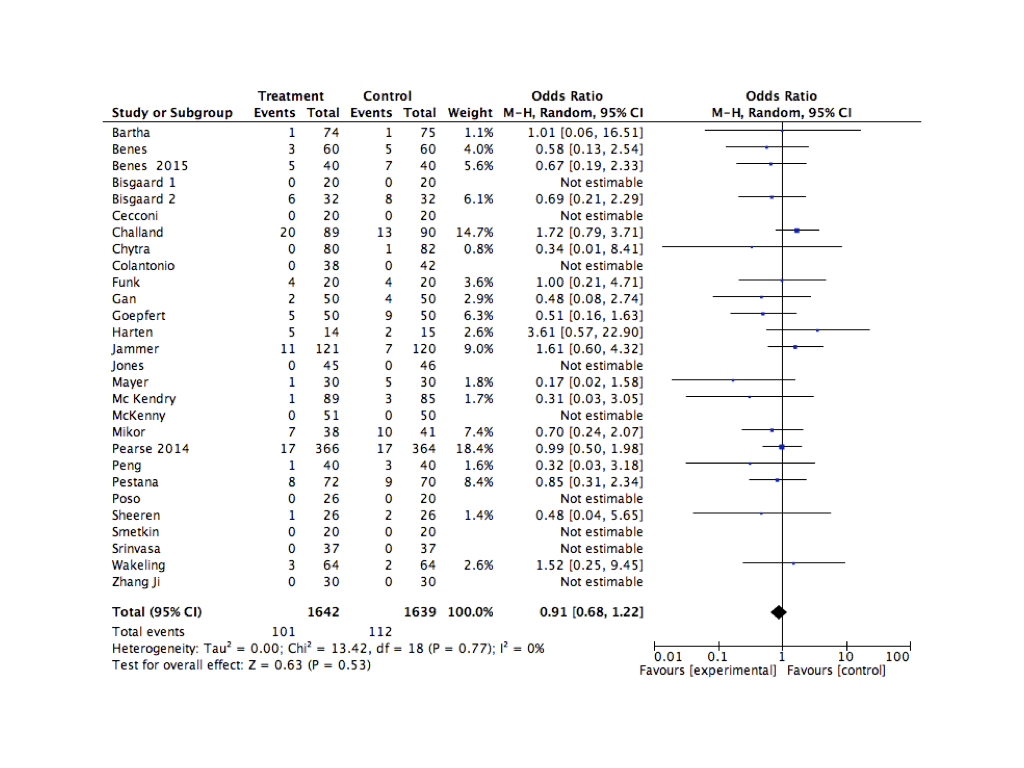

Supplement: Supplementary file 5 — Figure S4. Forest plot for postoperative acute kidney injury (AKI) (defined as the proportion of patients who developed postoperative worsening of renal function, whichever definition was used) including only those RCTs that showed a statistical difference between treatment versus control group during the perioperative period in the total amount of starch-based solutions (HES) administered. Size of squares for odds ratio reflects weight of trial in pooled analyses. Horizontal bars represent 95% confidence intervals. (TIFF 3072 kb) [file 13054_2019_2516_MOESM5_ESM.tiff]
