# Supplementary material for: Hemodynamic goal-directed therapy and postoperative kidney injury: an updated meta-analysis with trial sequential analysis
Source: Crit Care. 2019 Jun 26;23:232. doi: 10.1186/s13054-019-2516-4 (PMC6593609; doi:10.1186/s13054-019-2516-4)
Supplement: Supplementary file 7 — Table S1. Data concerning RCTs morbidity/mortality risk definition, population and type of surgery, tools and target used. (DOCX 23 kb) [file 13054_2019_2516_MOESM7_ESM.docx]

| **Author, Year, Country** | **Risk definition** | **Population** | **Surgery** | **Goals of Optimization** |
| --- | --- | --- | --- | --- |
| **Bartha et al ^17^**  2013, Europe | high | specific high-risk criteria | orthopedic | SV > 10%, DO_2_ > 600 L/min/m^2^ |
| **Bender et al ^18^**  1997, USA | low | No AMI, CABG, CHF | elective aortic and vascular | CI ≥ 2.8 L / min/m², 8 ≤ Pcwp ≤ 14 mmHg, SVR ≤ 1100 dyne∙sec/cm^5^ |
| **Benes et al ^19^**  2010, Europe | high | specific high-risk criteria | general abdominal | SVV <10%, CI > 2.5 L/min/m² |
| **Benes et al ^20^**  2015, Europe | moderate |  | orthopedic | PVI < 13% |
| **Berlauk et al ^21^**  1991, USA | low | exclusion of high-risk: no AMI, CABG, CHF | elective peripheral vascular | CI ≥ 2.8 L / min/m², 8 ≤ Pcwp ≤ 15 mmHg, SVR ≤ 1100 dyne∙sec/cm^5^ |
| **Bisgaard et al ^22^**  2013, Europe | high | specific high-risk criteria | peripheral vascular | rise of SVI < 10%, DO_2_ = 600 L/min/m^2^ |
| **Bisgaard et al ^23^**  2013, Europe | high | ASA III | abdominal aortic | rise of SVI 10%, DO_2_ = 600 L/min/m^2^ |
| **Bishop et al ^24^**  1995, USA | high | expected blood loss 2 L, major fractures requiring transfusion | emergent trauma | CI ≥ 4.5 L/min/m², DO_2_ ≥ 670 ml/min/m², VO_2_ ≥ 166 mL/min/m² |
| **Bonazzi et al ^25^**  2002, Europe | low | EF50% | elective vascular | CI = 3.0 L/min/m^2^, DO_2_ = 600 ml/min/m^2^, 10 Pcwp <18 mmHg, SVR> 1450 dyne∙sec/cm^5^ |
| **Boyd et al ^26^**  1993, Europe | high | specific high-risk criteria | emergent or elective  major abdominal or vascular | DO_2_ = 600 L/min/m^2^ |
| **Buettner et al ^27^**  2008, Europe | moderate | ASA II | major abdominal | SPV < 10% |
| **Cecconi et al ^28^**  2011, Europe | moderate | ASA II | orthopedic | SV < 10%, DO_2_ > 600 L/min/m^2^ |
| **Challand et al ^29^**  2013, Europe | high | ASAI-III,  age 60 yrs | major abdominal | SV increase 10% |
| **Chytra et al ^30^**  2007, Europe | high | multiple trauma expected blood loss 2 L | emergent trauma | SV optimization with FTc between 0.35 sec-0.4 sec |
| **Colantonio et al ^31^**  2015, Europe | moderate | ASA II-III | elective major abdominal | SVV < 15%  CI>2.5 mL/min/m^2^ |
| **Correa-Gallego et al ^32^**  2015, USA | high | ASA II-IV | elective liver resection | SVV < 2 DS of pre-induction |
| **Donati et al ^33^** 2007, Europe | high | ASA II-IV | elective major abdominal or aortic | O_2_ERe (SaO_2_ - ScvO_2_ /SaO_2_) =  27% |
| **Elgendy et al ^34^**  2017, Africa | moderate | ASA II-III | major abdominal | SVV < 125%  CI>2.5 mL/min/m^2^ |
| **Forget et al ^35^**  2011, Europe | moderate | ASA II-III | major abdominal | PVI < 13% |
| **Funk et al ^36^**  2015, USA | moderate | ASA II-III | major vascular | SVV< 15%  CI>2.5 mL/min/m^2^ |
| **Gan et al ^37^**  2002, USA | low | ASA I-III, blood loss 500 ml | elective general, urologic, gynecologic | SV optimization with FTc between 0.35 sec-0.4 sec |
| **Goepfert et al ^38^**  2013, Europe | high | ASA III | elective cardiac (on-pump) | SVV<10%,  CI =2 L/min/m^2^ |
| **Gomez-Izquierdo et al ^39^**  2017, Canada | low | ASAII | major abdominal | SVV<10%, |
| **Harten et al ^40^**  2008, Europe | high | ASA III | emergent abdominal | PPV<10% |
| **Jammer et al ^41^**  2010, Europe | moderate | ASA II | coloretal | ScVO_2_ >75% |
| **Jhanii et al ^42^**  2010, Europe | moderate | ASAII | elective gastro-intestinal | rise of SV <10% |
| **Jones et al ^43^**  2013, Europe | moderate | ASA II- III,  age 60 yrs | hepatic | rise of SV <10%,  CI > 3L/min |
| **Kapoor et al ^44^**  2008, India | moderate | Euroscore 3 | elective cardiac (on pump) | SVV> 10%, CI 2.5 and 4.2 L/min/m^2^, ScvO_2_ 70%, DO_2_ 450 and 600 ml/min/m^2^ |
| **Kaufmann et al ^45^**  2017, Europe | moderate | ASAIII | thoracic | SVV<10%,  CI =2.5 L/min/m^2^ |
| **Lai et al ^46^**  2015, Europe | moderate | ASA I-IV | major abdominal | SVV < 10%, |
| **Lobo et al ^47^**  2000, Brazil | high | age 60 years, previous disease of a vital organ | elective major abdominal or vascular | DO_2_ > 600 ml/min/m^2^ |
| **Luo et al ^48^**  2017, China | high | ASA III-IV | neurosurgical | SVV<15%,  CI =2.5 L/min/m^2^ |
| **Mayer et al ^49^**  2010, Europe | high | specific high risk criteria | major abdominal | CI > 2.5 L/min/m^2^ and SI >35 ml/m^2^ |
| **McKendry et al ^50^**  2004, Europe | low |  | elective cardiac (on-pump) | SVI >35 ml/m^2^ |
| **McKenny et al ^51^**  2013, Europe | moderate | ASA II | elective gynecological | SV <10% |
| **Mikor et al ^52^**  2015, Europe | high | age 60 years, ASA II-III | major abdominal and vascular | ScVO_2_ >75% |
| **Moppett et al ^53^**  2014, Europe | high | age > 80 years | orthopedic | SV increase < 10% |
| **Noblett et al ^54^**  2006, Europe | moderate | ASAII | colo-rectal surgery | SV optimization with FTc between 0.35 sec-0.4 sec |
| **Osawa et al ^55^**  2016, multicentric | high | specific high risk criteria | elective cardiac (on-pump) | CI > 3 L/min/m^2^ |
| **Pearse et al ^56^**  2005, Europe | high | specific high-risk criteria, POSSUM | elective or emergent major general | DO_2_ >600 ml/min/m^2^, SV increase < 10% |
| **Pearse et al ^57^**  2014, Europe | high | specific high-risk criteria, POSSUM | elective or emergent major general | SV increase < 10% |
| **Peng et al ^58^**  2014, China | low |  | orthopedic | SVV< 12% |
| **Pestana et al ^59^**  2015, Europe | high | specific high-risk criteria | elective major abdominal | CI >2.5 L/min/m^2^ |
| **Polonen et al ^60^**  2000, Europe | low | EF 50% | elective cardiac (on-pump) | SvO_2_ >70%, Lactate ≤ 2.0 mmol/L |
| **Poso et al ^61^**  2014, Europe | moderate | ASAII | bariatric | SVV< 12% |
| **Salzwedel et al ^62^**  2013, Europe | moderate | ASAII-III | elective major abdominal | PPV 10% and CI 2.5 L/min/m^2^ |
| **Sandham et al ^63^**  2003, Canada | high | age 60 years, ASA III-IV | elective or emergent major abdominal, thoracic, vascular, or orthopedic | CI 3.5 and 4.5 L/min/m^2^,550 DO_2_ 600 mL/min/m^2^, MAP 70 mmHg, Pcwp 18 mmHg |
| **Schmid et al ^64^**  2016, Europe | moderate | age 60 years | major abdominal | CI >2.5 L/min/m^2^ MAP 70 mmHg, GEDI> 640 ml/m^2^ |
| **Schereen et al ^65^**  2013, Europe | high | age 60 years, ASA III-IV | major abdominal and cystectomy | SVV 10% |
| **Shoemaker et al ^66^**  1998, USA | high | specific high-risk criteria | emergent or elective major abdominal | CI 4.5 L/min/m^2^, DO_2_ 600 mL/min/m^2^, VO_2_ 170 mL/min/m^2^ |
| **Smetkin et al ^67^**  2009; Europe | low | Euroscore2,  EF50% | elective cardiac (off-pump) | ITBI 850-1000 mL/m^2^, ScvO_2_ > 60 % |
| **Srinvasa et al ^68^**  2012, Australia | high | age 60 years  ASA II-III | elective colectomy | SV optimization with FTc between 0.35 sec-0.4 sec |
| **Valentine et al ^69^**  1998, USA | low | exclusion of high-risk | elective aortic | CI ≥ 2.8 L/min/m^2^, 8 ≤ Pcwp ≤ 15 mmHg, SVR ≤ 1100 dyne∙sec/cm^5^ |
| **Van Beest et al ^70^**  2014, Europe | high | age 60 years  ASA II-III | elective major abdominal | StO_2_ ≥ 80% |
| **Wakeling et al ^71^**  2005, Europe | low | POSSUM | elective major bowel | SV optimization and rise in CVP 3 mmHg |
| **Weinberg et al ^72^**  2017, Australia | high | age 60 years  ASA II-III | pancreaticoduodenectomy | CI ≥ 2. L/min/m^2^ and SVV > 20% |
| **Wenkui et al ^73^**  2010, China | high | age 60 years  ASA II-III | gastro-intestinal | Lactate 1.6 mmoL/L |
| **Wilson et al ^74^**  1999, Europe | high | coexisting medical conditions, POSSUM | elective major (abdominal, vascular, urologic) | DO_2_ 600 mL/min/m^2^ |
| **Wu al ^75^**  2017, China | low | ASAI-II, exclusion of high-risk | neurosurgical | CI ≥ 2.5 L/min/m^2^ and SVV 12% |
| **Xu al ^76^**  2017, China | low | ASAI-II, exclusion of high-risk | thorascopic lobectomy | CI ≥ 2.5 L/min/m^2^ and SVV 13% |
| **Zakaleva et al ^77^**  2013, Europe | moderate | ASA II-III | bowel resection | SV optimization with FTc between 0.35 sec-0.4 sec |
| **Zeng et al ^78^**  2014, China | moderate | ASA II-III | bariatric | 8 < SVV< 13% |
| **Zhang Jian et al ^79^**  2013, China | low | ASAI-II, exclusion of high-risk | thorascopic lobectomy | CI ≥ 2.5 L/min/m^2^ and SVV 10% |
| **Zhang Ju et al ^80^**  2012, China | low | ASA I-II | gastro-intestinal | PPV 11% |
| **Ziegler et al ^81^**  1997, USA | low | No AMI, CABG, CHF | elective vascular (aortic and limb salvage) | SvO_2_ ≥ 65%, Hb ≥ 10 g/dl, Pcwp ≥ 12 mmHg |

Table 1. Data concerning RCTs morbidity/mortality risk definition, population and type of surgery, tools and target used. Abbreviations: AMI: acute miocardial infarction; CABG: coronary-artery bypass grafting; CHF: chronic heart failure; ASA: American Society of Anesthesiologists; PPV : Pulse Pressure Variation; PVI : Pleth Variability Index; SVV : Stroke Volume Variation; SPV : Systolic Pressure Variation; SV: Stroke Volume; CI: Cardiac Index; MAP: Mean Arterial Pressure; CVP: Central Venous Pressure; SVI:Stroke Volume Index; SVRI:Systemic Vascular Resistance Index; ScvO_2_: Central Venous Oxygen Saturation; SvO_2_ Mixed Venous Oxygen Saturation, DO_2_; Oxygen Delivery; EVLWI: Extravascula Lung Water Index ;Pcwp: pulmonary capillary wedge pressure; PAC: pulmonary artery catheter; FTc: flow-time-corrected, O_2_ERe: Estimated Oxygen Extraction Ratio, SVR: Systemic Vascular Resistance, VO_2_: Oxygen Consumption, ITBI: Intra-Thoracic Blood Volume index, NTG :nitroglygerin; NTP:nitroprussiate; POSSUM: Physiological and Operative Severity Score for the enUmeration of Mortality and morbidity; GEDI: Global End-Diastolic Index; StO_2_ Tissue Oxygenation, EF: Ejection Fraction, SaO_2_: Arterial Oxygen Saturation.

In 12 studies hemodynamic monitoring was performed (18,21,24-26,47,60,63,66,69,74,81) with PAC, with oxygen delivery, cardiac output, mixed venous oxygen saturation and lactate as goal parameters. In 9 studies the LiDCO™ (17,22,23,40,43,46,53,56,57) (Lithium indicator Dilution Cardiac Output) system was used to measure cardiac output. In other 17 studies (19,28,31,32,34,36,44,48,49,58,61,65,72,75,76,78,79) a non invasive cardiac output measurement (FloTrac™), based on the analysis of arterial waveform, was performed. In 12 studies (29,30,37,39,45,50,51,54,61,68,71,77) an esophageal Doppler was used and stroke volume or the corrected flow time (i.e., the total amount of time the blood is traveling in a forward direction within the aorta corrected for heart rate; considered an index of systemic vascular resistance and sensitive to changes in left ventricular preload) guided hemodynamic optimization. In one study (33) the estimated oxygen extraction ratio, calculated as the ratio between the difference between arterial and central venous oxygen saturation (ScvO2) was the goal parameter. In 4 studies (27,38,64,67) the PiCCO™ plus system was used to analyze the arterial pressure curve to monitor hemodynamics. One study (59) used the NICOM™ (non invasive cardiac output monitoring based on biorectance) system to monitor cardiac output. Another study (35) used the Masimo™ set pulse oxymeter to monitor PVI. In two studies (41,52) the goal parameter was ScvO2 75%. One study (41) did not explicitly declare which system was used to monitor SV. In one study (62) the ProAQT™ monitor was used to measure CI. In one study hemodynamic optimization was guided by serial measurement of blood lactate levels (73). In another study (80) GDT was guided by pulse pressure variation, which was recorded using a Datex Ohmeda S/5 Monitor. Another study (20) used a continuous non-invasive blood pressure monitoring (CNAP®). In the last study (69) tissue oxygenation was monitored non-invasively by near infrared spectroscopy using the InSpectra System [Model 650; Hutchinson Technology, Hutchinson, MN, USA].
