# Supplementary material for: Hemodynamic goal-directed therapy and postoperative kidney injury: an updated meta-analysis with trial sequential analysis
Source: Crit Care. 2019 Jun 26;23:232. doi: 10.1186/s13054-019-2516-4 (PMC6593609; doi:10.1186/s13054-019-2516-4)
Supplement: Supplementary file 8 — Table S2. The risk of bias assessment for each trial, according to the Cochrane domain-based evaluation. (DOCX 17 kb) [file 13054_2019_2516_MOESM8_ESM.docx]

|  | | | | | |  |
| --- | --- | --- | --- | --- | --- | --- |
| **Author, Year, Country** | **Blinding of participant and personel**  **(performance**  **bias)** | **Random sequence generation**  **(selection bias)** | **Allocation concealment**  **(selection bias)** | **Outcome assessment**  **(detection**  **bias)** | **Incomple**  **outcome**  **data**  **(attrition bias)** | **Selective reporting**  **(reportig**  **bias)** |
| Bartha et al ^17^  2013, Europe |  | **+** | **+** | **+** | **+** | **+** |
| Bender et al ^18^  1997, USA | **-** | **-** | **-** |  | **-** |  |
| Benes et al ^19^  2010, Europe |  | **+** | **+** | **+** | **+** | **+** |
| Benes et al ^20^  2015, Europe |  | **+** | **+** | **+** | **+** | **+** |
| Berlauk et al ^21^  1991, USA | **-** | **-** | **-** | **-** | **-** |  |
| Bisgaard et al ^22^ 2013, Europe | **+** | **+** |  | **+** | **+** | **+** |
| Bisgaard et al ^23^  2013, Europe | **+** | **+** |  | **+** | **+** | **+** |
| Bishop et al ^24^  1995, USA | **-** | **-** | **-** | **-** |  | **+** |
| Bonazzi et al ^25^  2002, Europe |  | **+** |  | **-** | **-** | **-** |
| Boyd et al ^26^  1993, Europe | **-** | **-** | **-** |  |  |  |
| Buettner et al ^27^  2008, Europe |  |  | **+** | **+** |  |  |
| Cecconi et al ^28^  2011, Europe |  |  | **+** | **+** | **+** | **+** |
| Challand et al ^29^  2013, Europe | **+** | **+** | **+** | **+** |  | **+** |
| Chytra et al ^30^  2007, Europe | **-** | **-** |  | **+** | **+** | **+** |
| Colantonio et al ^31^  2015, Europe | **+** | **+** |  | **+** | **+** | **+** |
| Correa-Gallego et al ^32^  2015, USA | **+** | **+** | **+** | **+** | **+** |  |
| Donati et al ^33^  2007, Europe |  | **+** |  | **+** | **+** | **+** |
| Elgendy et al ^34^  2017, Africa | **+** |  |  | **+** | **+** | **+** |
| Forget et al ^35^  2011, Europe |  | **+** | **+** | **+** | **+** | **+** |
| Funk et al ^36^  2015, USA | **+** |  | **+** | **+** | **+** | **+** |
| Gan et al ^37^  2002, USA |  | **+** | **+** | **+** | **+** | **+** |
| Goepfert et al ^38^  2013, Europe | **+** | **+** | **+** |  | **+** | **+** |
| Gomez-Izquierdo et al ^39^  2017, Canada | **+** | **+** | **+** | **+** | **+** |  |
| Harten et al ^40^  2008, Europe |  |  | **+** | **+** | **+** | **+** |
| Jammer et al ^41^  2010, Europe |  | **+** | **+** | **+** | **+** | **+** |
| Jhanii et al ^42^  2010, Europe |  | **+** | **+** | **+** | **+** |  |
| Jones et al ^43^  2013, Europe | **+** | **+** | **+** |  |  |  |
| Kapoor et al ^44^  2008, India |  |  | **+** | **+** |  | **+** |
| Kaufmann et al ^45^  2017, Europe | **+** | **+** | **+** | **+** | **+** |  |
| Lai et al ^46^  2015, Europe | **+** | **+** | **+** | **+** | **+** |  |
| Lobo et al ^47^  2000, Brazil |  | **+** |  |  | **+** | **+** |
| Luo et al ^48^  2017, China |  | **+** |  | **+** | **+** |  |
| Mayer et al ^49^  2010, Europe |  |  | **+** | **+** | **+** | **+** |
| McKendry et al ^50^  2004, Europe |  | **+** | **+** | **+** | **+** | **+** |
| McKenny et al ^51^  2013, Europe |  | **+** | **+** | **+** | **+** | **+** |
| Mikor et al ^52^  2015, Europe | **+** | **+** |  | **+** | **+** |  |
| Moppett et al ^53^  2014, Europe | **+** | **+** | **+** | **+** | **+** | **+** |
| Noblett et al ^54^  2006, Europe | **+** | **-** | **+** | **+** | **+** | **+** |
| Osawa et al ^55^  2016, multicentric |  | **+** | **+** | **+** | **+** | **+** |
| Pearse et al ^56^  2005, Europe |  | **+** | **+** | **+** | **+** | **+** |
| Pearse et al ^57^  2014, Europe | **+** | **+** | **+** | **+** | **+** | **+** |
| Peng et al ^58^  2014, China |  | **+** |  | **+** | **+** |  |
| Pestana et al ^59^  2015, Europe | **+** | **+** | **+** | **+** | **+** |  |
| Polonen et al ^60^  2000, Europe |  |  | **+** | **+** | **+** | **+** |
| Poso et al ^61^  2014, Europe |  | **-** | **-** | **+** | **+** | **+** |
| Salzwedel et al ^62^  2013, Europe | **+** | **+** | **+** | **+** | **+** | **+** |
| Sandham et al ^63^  2003, Canada | **+** | **+** | **+** | **+** |  | **+** |
| Schmid et al ^64^  2016, Europe | **+** | **+** | **+** | **+** |  | **+** |
| Schereen et al ^65^  2013, Europe |  |  | **+** | **+** | **+** | **+** |
| Shoemaker et al ^66^  1998, USA |  | **-** | **-** | **-** | **-** | **+** |
| Smetkin et al ^67^  2009; Europe | **-** | **-** | **-** | **-** | **+** | **+** |
| Srinvasa et al ^68^  2012, Australia | **+** | **+** | **+** | **+** | **+** |  |
| Valentine et al ^70^ 1998, USA |  |  | **+** | **+** | **+** | **+** |
| Van Beest al ^71^  2014, Europe | **+** | **+** |  | **+** | **+** |  |
| Wakeling et al ^72^  2005, Europe |  | **+** | **+** | **+** | **+** | **+** |
| Weinberg et al ^73^  2017, Australia | **+** | **+** | **+** | **+** | **+** | **+** |
| Wenkui et al ^74^  2010, China |  | **+** | **+** | **+** | **+** | **+** |
| Wilson et al ^74^  1999, Europe | **+** | **+** | **+** | **+** | **+** |  |
| Wu et al ^75^  2017,China, |  |  |  | **+** | **+** |  |
| Xu et al ^76^  2017, China | **+** | **+** | **+** | **+** | **+** | **+** |
| Zakaleva et al ^77^  2013, Europe |  | **+** | **+** |  | **+** | **+** |
| Zeng et al ^78^  2014, China |  |  |  |  | **+** | **+** |
| Zhang Jian et al^79^  2013, China |  | **+** | **+** |  | **+** | **+** |
| Zhang Ju et al ^80^  2012, China |  | **+** |  |  | **+** | **+** |
| Ziegler et al ^81^  1997, USA |  | **-** | **-** | **+** |  |  |

Table 2. The risk of bias assessment for each trial, according to the Cochrane domain-based evaluation. This is a two-part tool, addressing seven specific domains (namely sequence generation, allocation concealment, blinding of participants and personnel, blinding of outcome assessment, incomplete outcome data, selective outcome reporting and ‘other issues’) that are strongly associated with bias reduction. The green plus indicates low risk of bias, the red minus indicates high risk of bias, the white colour indicates unclear risk of bias.(see text for details)
